# Down-conversion *en-face* optical coherence tomography

**DOI:** 10.1364/BOE.10.000772

**Published:** 2019-01-23

**Authors:** Adrian Podoleanu, Ramona Cernat, Adrian Bradu

**Affiliations:** Applied Optics Group, School of Physical Sciences, University of Kent, Canterbury CT2 7NH, UK

## Abstract

We present an optical coherence tomography (OCT) method that can deliver an *en-face* OCT image from a sample in real-time, irrespective of the tuning speed of the swept source. The method, based on the master slave interferometry technique, implements a coherence gate principle by requiring that the optical path difference (OPD) between the arms of an imaging interferometer is the same with the OPD in an interrogating interferometer. In this way, a real-time *en-face* OCT image can originate from a depth in the sample placed in the imaging interferometer, selected by actuating on the OPD in the interrogating interferometer, while laterally scanning the incident beam over the sample. The generation of the *en-face* image resembles time domain OCT, with the difference that here the signal is processed based on spectral domain OCT. The optoelectronic processor operates down-conversion of the chirped radio frequency signal delivered by the photo-detector. The down-conversion factor is equal to the ratio of the maximum frequency of the photo-detected signal due to an OPD value matching the coherence length of the swept source, to the sweeping rate. This factor can exceed 10^6^ for long coherence swept sources.

## 1. Introduction

Master Slave (MS) OCT method was introduced to address the problems raised by using a Fourier transformation in processing the signal proportional to the spectrum modulation at the interferometer output. In the initial report [1] introducing the MS operation, an interrogating interferometer was presented, and a comparison operator of the electrical signals delivered by the spectrometers or photo-detectors at the outputs of the imaging and interrogating interferometers. The MS method was evolved to a more practical procedure where the interrogating interferometer was replaced by the same imaging interferometer employed sequentially in two steps, Master and Slave. At the Master stage, a mirror is employed as sample and channeled spectra acquired for several OPD values are stored as masks. At the Slave step, when the sample replaces the mirror, the masks are compared with the acquired channeled spectrum. The comparison operation was performed digitally, via cross-correlations or matrix multiplications [2] and several advantages of the MS methods have been reported, such as: (i) direct production of *en-face* OCT images (with no need to acquire the whole volume followed by software cut) [3], (ii) elimination of the need of resampling, and (iii) tolerance to dispersion in the interferometer [4], that makes the MS ideally suited to be used in conjunction with coherence revival [5].

In initial MS reports, the masks were experimentally recorded channeled spectra obtained using the same Slave interferometer with a mirror as sample, at the Master stage. This meant that the masks retained the envelope of the swept source spectrum as well as their decaying amplitude with OPD. This deficiency was eliminated in last reports on the MS by the introduction of the complex Master Slave (CMS) method [6]. This allows to theoretically infer any number of masks using only two or more experimentally acquired channeled spectra. The masks so calculated are deprived from noise and all exhibit the same maximum amplitude. In addition, they are complex, allowing access to phase and calculation of real and imaginary parts of the degree of similarity between the mask and the signal. This leads to better stability for the final result in respect to subwavelength variation of the OPD, similar to that provided by an I&Q demodulator. CMS was subsequently used for producing a combined OCT with scanning laser ophthalmoscopy (SLO) instrument for the eye [7], imaging basal cell carcinoma of eye lids [8], Gabor fusion [9] and applied to embryology [10]. The configuration with two interferometers was only utilized in the teaching of the MS principle and deemed initially not practical for two reasons: need for a second interferometer and delivering a single depth at a time, like in *en-face* time domain OCT [11,14].

In this report, the originally proposed MS configuration, with two physical interferometers is employed in practice for the first time. We demonstrate that such a configuration presents unique advantages in terms of signal processing and can complement conventional detailed OCT investigations, either performed using the conventional fast Fourier transform (FFT) or Master Slave principle using multiple masks as reported so far. In this paper, an optoelectronic processor replaces the digitizer used by the MS-OCT in all previous reports. Selection of depth at which an *en-face* image is produced, is performed via the OPD change in a separate, physical interferometer. Such a procedure can be executed continuously, in real-time while the incident beam is laterally scanned over the target and the image delivery is instantaneous. In addition, we draw attention here to an exquisite property of the original MS configuration, not considered so far. The comparison part of the optoelectronic processor operates as a downconverter, which brings advantages in terms of processing speed and cost.

We present here two solutions for the down-conversion, either based on analogue mixing or on conventional digitization. We evaluate the impact of down-conversion on the sensitivity decay and axial resolution versus the OPD, in comparison with the fully functional CMS-OCT configuration using a single interferometer and a digitizer. The utilization of a mixer was already proposed in a previous report [12], where the amplitude of a single frequency band was extracted from the photo-detected signal while tuning the optical frequency of the optical source, by mixing the photo-detected signal with a reference signal of a particular chosen frequency delivered by a local oscillator. However, the method reported is only applicable to a swept source OCT system using a linear swept laser source, which provides a highly linear dependence between optical frequency and time sweep.

## 2. Methodology

### 2.1 Experimental set-up

**Fig. 1:**
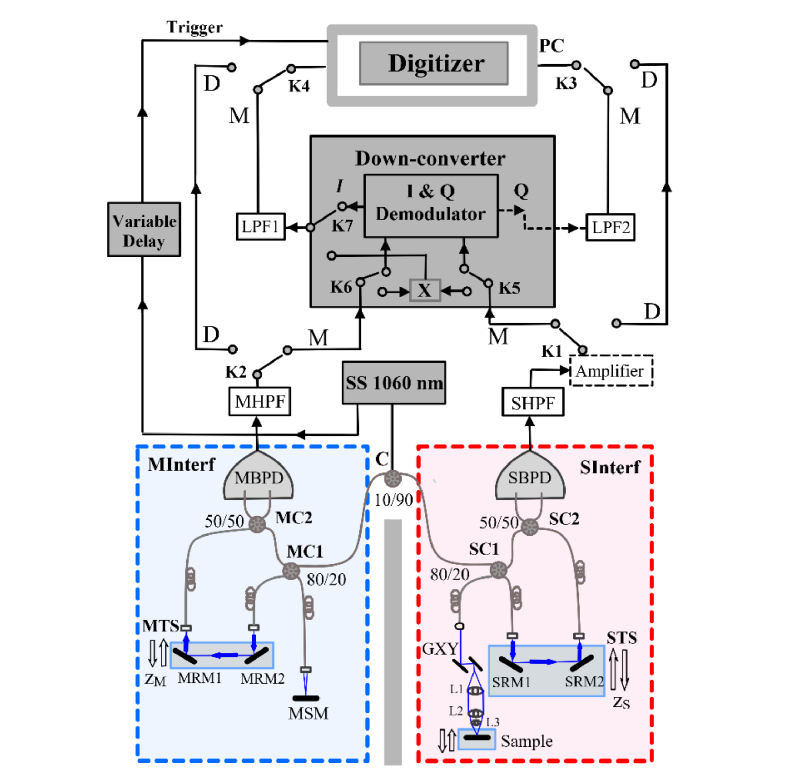
Experimental set-up. SC1, SC2, MC1, MC2: directional fiber couplers; SBPD, MBPD: balanced photo-detectors; STS, MTS: translation stages; SRM, MRM, MSM: metallic flat mirrors; MHPF, SHPF: high pass filters; LPF1, LPF2: low pass filters; M: M-method, D: D-method.

The system employed for this study is schematically presented in Fig. 1

. This represents a swept source (SS)-OCT implementation of the MS principle. For the present study, a swept source, from Axsun Technologies, 1050 nm central wavelength, operating at 200 kHz, over a tuning bandwidth of 111 nm (982-1093 nm) at 12 dB was employed [13].

The light at the output of a swept source, SS, is conveyed by a 10:90 fiber coupler (C) towards two interferometers. SInterf (red dashed rectangle in Fig. 1) represents the measuring/imaging interferometer, or Slave according to [1] whilst MInterf (blue dashed rectangle) represents the interrogating interferometer, or Master, according to the same reference [1]. Both interferometers consist of similar directional couplers SC1 and MC1, that direct 20% of the input power towards the sample, respectively mirror MSM and 80% towards a reference arm. To adjust the OPD in the reference path of MInterf, two mirrors MRM1 and MRM2 are placed on a translation stage (MTS). In the SInterf, mirrors SRM1 and SRM2 are also placed on a translation stage STS. In the sample arm of SInterf, a pair of orthogonal galvanometer scanners, GXY is used to scan the beam over the sample. Light waves from the reference and sample arms in each interferometer are combined in 50:50 directional fiber couplers SC2 and MC2 respectively. The outputs of these feed two identical balanced photo-detectors SBPD and MBPD (Thorlabs, Model PDB481C, AC 30 KHz −1 GHz). The photo-detected signal out of SBPD in the SInterf is further amplified by a Mini-Circuits amplifier (ZHL-32A + , 0.05 to 130 MHz). This is shown in dashed line, as it will be used for weak reflecting samples only, as detailed below. Two similar high pass filters (SHPF, MHPF), with a cut-off frequency of 6.7 MHz (Model EF513 – Thorlabs) are used to eliminate the DC and the low frequency variation of the photo-detected signal from both interferometers, before being multiplied either using mixing (M-method) or digitally (D-method). The signals from the two interferometers are sent via switches K1 and K2, K3 and K4 to either to and from the Down-converter block or, directly to a dual input digitizer placed in a PC. The Down-converter block employed either a mixer, X, or an I&Q demodulator, via switches K5, K6 and K7. After digitization, data is processed using a LabVIEW (National Instruments, Austin, Texas) in-house software. It should be noticed that an ultrafast digitizer, (Alazartech, Quebec, Canada, model ATS9360), capable of sampling data at rates as high as 1.8 GS/s is employed. As described below, this was used for sampling at maximum sampling rate in the D-mode and CMS-mode and at rates from 100 MS/s to the maximum sampling rate in the M-mode.

To sample the correct time interval relative to the trigger signal from the swept source, a pulse function generator was used (Agilent Technologies 81160A), in the block Variable Delay, to adjust the start of the time interval sampled between zero to several microseconds.

To scan the beam over the Sample, a dual galvanometer scanning head, GXY (Cambridge Technology 62xxH Series Galvanometer Scanners,) is used. As in time domain OCT using lateral priority scanning [14], the bandwidth needed is comparable to the inverse of the time required to move the beam from a pixel to the next, i.e. similar to that in raster or TV display, or that in *en-face* scanning in confocal microscopy.

### 2.2 The M-method

When using the M-method the two analogue signals delivered by the two photo-detector units are applied to a Downconverter block, based on mixing the two signals. To prove the possibility to perform MS operation using analogue mixing, this is implemented using either a mixer (two inputs one output) or an I&Q demodulator (two inputs, two outputs, that consists in two mixers).

Considering that the maximum frequency in the channeled spectrum is F_max_ and the maximum frequency to produce an image f_im_, the down-conversion factor is DCo = F_max_/f_im_. Normally, to reduce noise, the imaging bandwidth is restricted to that of allowing sufficient time to display each pixel, which is equal to the sweeping time. Let us consider the source, that sweeps in 5 μs, with a duty ratio of η ~60%, i.e. the useful spectrum is tuned within 1/(ηF_S_) = 3 μs, where F_S_/η = F’_S_ defines an equivalent sweeping frequency. This gives F’_S_ = 333 kHz, so the digitizer would need to sample at least 666 kHz, and f_im_ = F’_S_.

The number of resolvable points in depth is obtained by AR/ARI, where AR is the axial range and ARI the axial resolution interval. AR = 0.25λ^2^/δλ = bλ^2^/δλ. ARI = aλ^2^/Δλ, where a for a Gaussian spectrum is a = 2ln2/π and for a top hat spectrum, typical for a swept source, a = 0.5 [15,16]. So, AR/ARI = (b/a) Δλ/δλ. This also corresponds to the approximate number of cycles in the channelled spectrum. The maximum frequency in the photo-detected spectrum becomes: F_max_ = F’_S_ × AR/ARI. This means that the down-conversion factor DCo = F_max_/f_im_ = AR/ARI. This ratio determines how much larger the frequency of the photo-detected signal is in respect to the equivalent sweeping rate. In other words, using the M-method, the signal to be processed has a frequency that is DCo times smaller than the frequency of the signal handled by conventional OCT technology.

In the M case, the digitizer processing the signals from the Down-converter needs to digitise signals at a frequency proportional to the inverse time of equivalent sweeping, 1/F’_S_. For the 200 kHz swept source used, sufficient strong interference signal could be obtained from an OPD = AR ~3 cm, where the RF spectrum of the photo-detected signal extends to over 1.1 GHz. Using a value of ARI ~13 μm experimentally measured at FWHM, gives a DCo ~2x10^3^. A similar value for the DCo is obtainable by dividing the respective RF value corresponding to an OPD reaching the AR, to the F’_S._ However, if a swept source would be used, similar to that reported in [17], with over 200 m extra-long coherence length, a DCo > 10^7^ could be achieved.

The method M presents an immediate gain in terms of digitizer cost. The extra components needed are a balanced photodetector unit, two couplers and a mixer or an I&Q demodulator, in total costing much less than the cost of a fast digitizer such as from Alazartech or National Instruments, whose cost can approach or exceeds $10K. The choice of mixer or of an I&Q demodulator determines different down-conversion modes of operation of the set-up in Fig. 1.

### 2.3 The I&Q demodulator

The I&Q demodulator (Mini-Circuit, model ZFMIQ-10D), operating within the frequency range 9-11 MHz, implements quadrature demodulation, consisting in two mixers, where one of the mixers operates with one of the signals shifted by 90°. As the phase shift cannot be implemented in a wide bandwidth, such a demodulator exhibits a narrow bandwidth. The signal outputs of the two mixers, I(0°) and Q(90°) are sent to two low pass filters (ILPF, QLPF) (Stanford Research, Low Noise Preamplifier Model SR560) whose outputs are directed to the two inputs of the dual input digitizer. Two signals, Q and I are produced and for completion of quadrature demodulation, the digitizer implements [Q^2^ + I^2^]^1/2^ as a brightness measure for each lateral pixel displayed, based on a LabVIEW program, LabV/IQ. This program incorporates a program to deliver *en-face* images in synchronism with the signals driving the scanning head.

### 2.4 The broadband mixer

Another possibility is that of using a simple mixer, such as Mini-Circuit, ZFM-4, 5-1250 MHz). This consists of two transformers and a bridge of 4 diodes, and such configurations can cover large bandwidths, exceeding tens of GHz. In Fig. 1, when the Down-converter employs a mixer, there is a single output and hence a single low pass filter is needed and a single input of the digitizer is used in this case. Interference amplitude is measured using a LabVIEW program, LabV/BM.

### 2.5 The D-method

D-method involves digitisation of the two photo-detected signals delivered by the two photodetector units SBPD and MBPD and implementing the comparison operation in digital format. In Fig. 1, the 4 switches, K1, K2, K3 and K4 are switched to position D. Given the explanation above on the difference between the MS and CMS protocols, it is expected that the D-method should be equivalent to the MS operation implemented using experimentally acquired masks. The MS method delivered slightly worse sensitivity and resolution than achievable using the improved protocol, of CMS [6]. The same CMS algorithm is used here for calculation of the interference strength using a LabVIEW Program, LabV/D. To produce *en-face* OCT images using the D-method, the master stage involving the use of a reflective object as a sample is not required. The mask T(k,z) to generate an *en-face* image is delivered by the slave interferometer, for which reason both channels of the digitizer are engaged. To produce the brightness (*R*) of a particular pixel in the image, the product between the channelled spectrum, CS, delivered by the slave interferometer and the channelled spectrum delivered by the master, needs to be computed:

R(z)=T(k,z)⋅CS(k). (1)

The *en-face* image corresponding to a specific axial position is selected by adjusting the OPD in the Master interferometer. When using CMS, the complex form of the two signals is manipulated with calculation of real and imaginary parts. This confers stability to the output signal, similar to that obtained by using an I&Q demodulator. The difference is here that the Masks are replaced by instantly delivered electrical signals while sweeping the swept source frequency. An immediate disadvantage of the D-method is that a two inputs high speed digitizer is still needed and the Mask generated is affected by the envelope of the swept source spectrum.

### 2.6 Potential advantage of real time generation of the mask signal

The use of physical interferometers has the advantage, in either the M or D regime, of a potentially better tolerance to fluctuations in the swept source emission than that of the conventional CMS implementation using software generated masks. As the mask is produced in real time, i.e. at the time of measurement, stability in time of the mask signal is not required. Both conventional methods using FFT processing as well as the MS (or CMS) method using stored masks require sufficient stability in the swept source emission. Experiments have shown that in time, new masks must be produced, although such time can be very long. This is the case for other calibration methods of SS-OCT set-ups as well, variation of calibration in time is not typical to the MS protocol. As the two interrogating interferometers are driven by the same optical source, whatever fluctuation the optical source may exhibit in its tuning curve over time affects both interferometers. The mask here is produced at the time of measurement, delivered by the interrogating (Master) interferometer, so any variation in the SS nonlinearity sweeping is automatically compensated for.

### 2.7 Theory

Let us consider the swept source tuning the wavenumber g from a minimum wavenumber g_m_ to a maximum wavenumber g_M_ in a tuning bandwidth:

$$\Delta g=g_{M}-g_{m}.$$ (2)

Then in time, during a sweep duration T, the wavenumber varies as:

$$g=\gamma t+g_{m}$$ (3)

where the tuning is generally nonlinear, i.e. the slope *γ* varies in time. Let us consider initially the Slave interferometer equipped with a mirror as sample, i.e. both interferometers, each use two mirrors. The interference signal at the Slave and at the Master interferometer output can be respectively written as:

$$CS_{S}=A_{S}\cos\left[gz_{S}+h_{S}(k)+\theta_{s}\right]\;\text{and}\;CS_{M}=A_{M}\cos\left[gz_{M}+h_{M}(k)+\theta_{M}\right]$$ (4)

where *A_S_* and *A_M_* are the interference amplitudes, $z_{S}$ and $z_{M}$ optical path differences, $h_{S}$ and $h_{M}$ dispersion left uncompensated and $\theta_{s}$ and $\theta_{M}$ random phases in the slave and respective master interferometers. The factors *gz_S_* and *gz_M_* determine the pulsations of the photo-detected signals that are input to the down-converter. Let us consider, that the down-conversion is performed by a mixer, as the I&Q demodulator provides after processing the two output signals a similar operation, but with better stability to phase. Within each sweeping time T, the signal at the mixer output is proportional to the products of signals at the two mixer inputs:

$$s=A_{S}A_{M}\cos\left[gz_{S}+h_{S}(k)+\theta_{S}\right]\cdot \cos\left[gz_{M}+h_{M}(k)+\theta_{M}\right]$$ (5)

The equation above can be re-written as:

$$s=\frac{1}{2}A_{S}A_{M}\left\{\cos\left[g\Delta z+\Delta h+\Delta \theta \right]+\cos\left[g(z_{S}+z_{M})+h_{S}(k)+h_{M}(k)+\theta_{S}+\theta_{M}\right]\right\}$$ (6)

Here $\Delta z=z_{S}-z_{M}$ represents the differential OPD, $\Delta h=h_{S}(k)-h_{M}(k)$ is the variation of dispersion and $\Delta \theta =\theta_{S}-\theta_{M}$ is the variation of random phase, each calculated between the two interferometers. The first term varies slowly in time and it is the useful term retained by low pass filtering, while the second term has a large frequency variation and is eliminated by the low pass filtering. Because *g* is the same in both interferometers, even if *g* varies with time via the slope γ, signal for Δ*z* = 0 is selected only. Put it differently, when$z_{S}=z_{M}$, the pulsation frequency of the two photo-detected signals, *gz_S_* and *gz_M_* is the same and therefore their beating frequency is zero irrespective of the value of such pulsation frequency. A low pass filter with a time constant T will deliver:

$$s=\frac{0.5A_{S}A_{M}}{T}\int_{0}^{T}\cos\left(g\Delta z+\Delta h+\Delta \theta \right)dt$$ (7)

where *g*, Δ*h* and Δ*θ* are time variables. Let us consider for simplicity that dispersion is compensated, either in both interferometers, or matched in the two interferometers, ignore the random phase and consider a linear variation of *g* in time, i.e. γ is a constant. In this case, using Eq. (2), Eq. (6) becomes:

$$s\approx \frac{0.5A_{S}A_{M}}{T}\cdot \frac{2\sin\left(\frac{\gamma T\Delta z}{2}\right)\cos\left[\left(\frac{\gamma T}{2}+g_{m}\right)\Delta z\right]}{\gamma \Delta z}$$ (8)

The *≈* symbol reflects the approximations listed above. When Δz approaches zero, i.e. for the same OPD in both interferometers, a maximum is recorded,

$$s_{M}=0.5\;A_{S}\;A_{M}$$ (9)

It can be noticed that the first zero of the expression in Eq. (9) takes place for:

$$\Delta z=\frac{2\pi }{\gamma T}=\frac{2\pi }{\Delta g}$$ (10)

which defines a similar coherence gate width, as for the slave interferometer.

## 3. Results

### 3.1 Performance characterization

In implementing the configuration in Fig. 1 it was noticed that even using the band-pass filters, residual pulses due to the intensity modulation of the swept source emission would traverse to the outputs of the mixers. These create a bias in the image, even when using signal from one interferometer only, obscuring the coherence gate effect, reducing the contrast and contributing to noise. Therefore, irrespective of the method used, a trigger from the swept source was employed to control the sampling duration processed by the digitizer. Adjusting the delay on the trigger, and reducing the length to less than 3 µs, diminished the contribution of the amplitude pulsation during tuning.

**Fig. 2:**
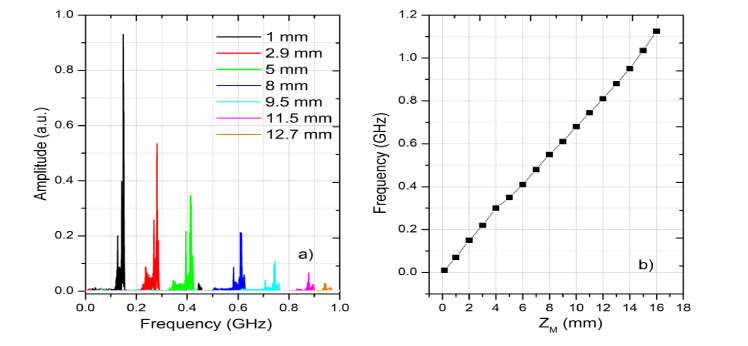
(a) RF spectrum of the photo-detected signal acquired with the Master Interferometer using the 200 kHz SS for 7 different values of the position, z_M_/2, of the translation stage, MTS. (b) Variation of the maximum frequency in the spectrum of the photo-detected signal with OPD.

Important for the down-conversion operation is the spectrum variation of the photo-detected signal with OPD. This is shown for the 200 kHz SS in Fig. 2(a)

, where the spectrum of the photo-detected signal in the MInterf is displayed versus the displacement of the translation stage MTS measured from OPD = 0. This shows the spread of frequencies that increases with OPD = z*_M_* (the values in the inset represent OPD/2). Considering the maximum frequency in the spectrum, its variation with OPD is displayed in Fig. 2(b). Operation of the two interferometers configuration is assessed for both M and D methods described above. Assessment refers to the axial resolution and decay of sensitivity with OPD. While the main interest here is to quantify the down-conversion of the RF spectrum of the photo-detected signal, experiments are also preformed using the conventional CMS method employing a digitizer and stored masks acquired using the Slave interferometer, for comparison. The results using the CMS method should be interpreted as similar to those using the conventional FFT signal processing after resampling of data. The graph in Fig. 2(b) suggests a conversion factor of 68 MHz/mm.

### 3.2 M-method

Depending on the type of Down-converter used, different protocols of scanning the sample in depth are possible. Using the Broadband Mixer, the OPD_M_ in the MInterf is varied, while when using the I&Q demodulator, the OPD in the SInterf, as detailed below.

Using the Broadband Mixer, different depths in the sample are selected by varying the OPD_M_ in the MInterf, observing the coherence gate condition: |OPD_M_ – OPD_S_| = |ΔOPD| = 0. Considering the bandwidth of the Broadband Mixer, 5-1250 MHz, this corresponds to a variation in the OPD_M_ that can be evaluated considering the variation of the dominant frequency in the RF spectrum of the photo-detected signal versus OPD. The maximum frequency of the photo-detected signal depends on the product of sweeping frequency by the OPD. Considering the 200 kHz SS and conversion factor vs OPD as 34 MHz/mm measured experimentally using the spectrum analyzer, the Broadband mixer can be used for a range of OPD values between 5/34 = 0.147 mm and 36.7 mm. This is well above the limit due to the digitizer, as detailed below. For a sample of optical thickness d_Max_, the length of the reference arm in the Master interferometer needs to be changed by 2d_Max_ to explore the sample from top to bottom. Using the I&Q demodulator, its bandwidth is limited to 9-11 MHz. There are two points to make:

(i) The spectrum of the two photo-detected signals needs to be maintained within the I&Q demodulator bandwidth. Let us choose an OPD adjusted in the interrogating (Master) interferometer to generate a photo-detected signal pulsating at the central frequency of the I&Q demodulator band ~10 MHz. The Master interferometer MInterf dictates the OPD value in the Slave interferometer SInterf where signal is acquired from, i.e. satisfying the coherence gate condition: |OPD_M_ – OPD_S_| = |ΔOPD| = 0. This means that in order to select other depths within the sample, the reference path length in the Slave interferometer needs to be changed, to keep the overall OPD_S_ in the Slave interferometer equal to the OPD_M_ in the Master interferometer. For a sample of optical thickness d_Max_, the reference arm length in the SInterf needs to be changed by 2d_Max_ to explore the sample from top to bottom. Considering a sample composed from a succession of reflecting layers, by varying the length of the reference arm in the Slave Interferometer, different layers are brought into the coherence gate, that always correspond to the same OPD_M_. In this way, the frequency of the signals sent to the two inputs of the Down-converter are similar. This limits the OPD values that can be interrogated to those within the range in thickness from top to that depth that determines the fixed frequency used. An immediate interesting conclusion is that down-conversion is obtained with constant sensitivity, where no dependence of sensitivity to the OPD is obtained, and this would be similar to time domain OCT. In time domain, irrespective of length of the sample arm, sensitivity is the same [18], measured in OPD = 0 by matching the reference arm length to the sampling arm length. The only variation is determined by the attenuation of light in the sample. However, when we say constant, this would be the sensitivity value at the OPD determining a 10 MHz modulation. At larger OPD values, the sensitivity will be lower. Another important conclusion is that if multiplexing is considered with several Master Interferometers, narrow Down-converters with different central frequency may be more suitable.
(ii) If the Down-converter exhibits a narrow bandwidth, then its bandwidth should be still sufficiently wide to cover the spread of frequencies due to the chirp. We have measured the spread of frequencies in the channeled spectrum. For instance, using the 200 kHz SS, an OPD = 0.29 mm, the maximum period in the sweep is 100 ns and the minimum period is 90 ns. This determines a spread of frequencies from 10 MHz to 11.1 MHz, just sufficient to fit the band of the I&Q demodulator used, ZFMIQ-10D, recommended for 9-11 MHz.

### 3.3 Experimental results

**Fig. 3:**
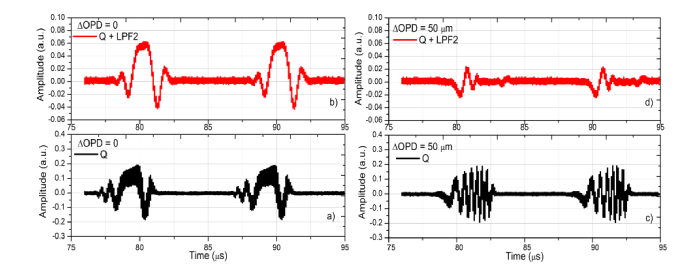
Signal amplitude at the Q output of I&Q demodulator before and after the low pass filter LPF2 for: a) ΔOPD = 0 and b) ΔOPD = 50 µm.

Essential in the operation of the configuration in Fig. 2 is that pulses due to the intensity modulation of the laser emission, that are present irrespective of the interference, to not be employed in the final calculation of the signal. The effect of using a 1 MHz low pass filter at the Q output of the I&Q demodulator is shown in Fig. 3

for OPD = 0 (left) and for OPD = 50 μm (right). In each figure two temporal evolutions are shown, of the Q output (bottom) and of the same signal at the low pass filter (LPF2) output (top), using no amplification. The signals at the bottom of both figures, at the Q-output, show thick traces. This is due to the fast oscillating signal at ~10 MHz (not possible to be resolved at the scale of Fig. 3) crossing residually from the Master interferometer. This superposes the useful signal expected from the demodulator, but it is completely eliminated by the low pass filter at its output. Figure 3 illustrates the operation of the coherence gate, with a large signal selected in Fig. 3 left and the difference of frequency the demodulator output, due to different OPD values in the two interferometers, as shown in Fig. 3 right for ΔOPD = 50 μm, seen on the Q output, attenuated at the LPF2 output.

A first observation is that the LPF2 output introduces a delay of 2.4 μs. A second observation is that the LPF2 enlarges slightly the transitions according to its inverse cut-off frequency. This illustrates the need to carefully control the time interval sampled and slightly reduce it below the duration of the laser emission. This is equivalent with processing a smaller bandwidth, that leads to some reduction in the axial resolution as documented below. Suitable adjustment of the length of time interval sampled, for less than 3 μs, is obtained by reducing the number of points sampled by the digitizer whilst its delay is controlled using the Variable Delay block shown in Fig. 1. These adjustments are experimentally performed, by moving away from ΔOPD = 0 by more than 100 μm, and optimizing the length and the delay for minimum output signal displayed by the digitizer. Figure 3 does not show it, but some of low frequency components, within the low pass filter band, mainly due to the laser emission intensity modulation, are not sufficiently reduced by the high pass filters, at 6.7 MHz. However, these components can be totally eliminated for larger OPD values, i.e. when using larger frequency I&Q demodulators and mixers, where much higher frequency cut-off high pass filters can be employed relative to the F’s (the I&Q demodulator used here operates at a relatively low frequency, 9-11 MHz, at the bottom of the RF spectrum in Fig. 2).

The acquisition parameters of the digitizer for the Method D and CMS are different from those when the digitizer is used for the M-method. Not only that the sampling rate is maximum, 1.8GS/s for the D and CMS method, but the delay used is readjusted to less than 1 μs.

### 3.4 Variation of sensitivity with OPD

**Fig. 4:**
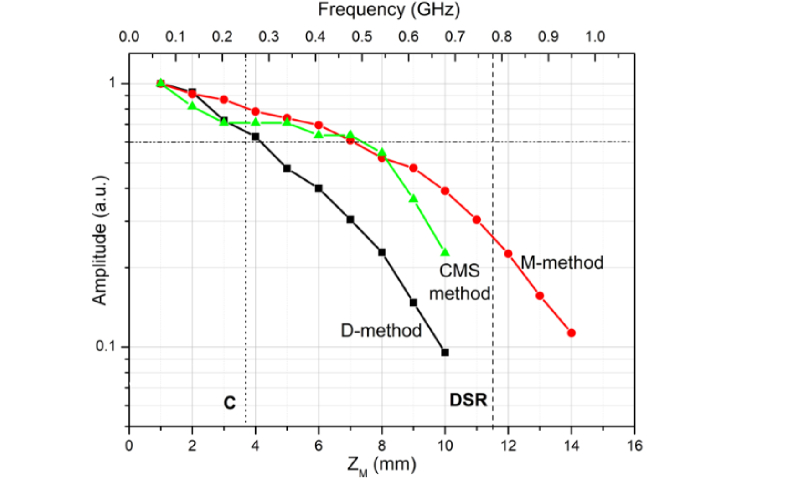
Signal amplitude versus z_M_ for all methods: M with the Broadband Mixer, D and CMS.

Signal amplitude versus OPD was measured to evaluate the sensitivity variation. The results presented in Fig. 4

, were produced by varying the OPD in the MInterf and matching the OPD in the SInterf until a maximum signal was obtained. The OPD value is double the value Z_M_ shown, measured using the displacement of the translations stage MTS. It is known that the larger the OPD, the smaller the strength of interference signal due to the limited dynamic coherence length of the swept source. When using the Broadband Mixer, by changing OPD_M_, it is expected that similar decay is obtained as that already measured in [1], where the mask stored corresponded to experimentally measured channeled spectra.

The curves obtained for the M, D and CSM methods are shown in Fig. 4 and display the expected decay of sensitivity with OPD, due to reduction of the overlap of the two wave-train lengths corresponding to the two interfering waves. All three curves exhibit decay of sensitivity with OPD as expected. Not only that the decay for the M-method is less than using the other two methods, but significant signal is obtained beyond the limits established by conventional technology. A first constraint is that of the clock signal of the 200 kHz swept source, pulsating at 500 MHz. This limits the axial range to the OPD value that creates a modulation of 250 MHz according to Nyquist theorem, shown by the vertical dotted line at 3.67 mm depth, marked as C. A second limit is that of the digital sampling rate (DSR) of the digitizer. At maximum 1.8 GS/s, experimentally, using FFT, we noticed a reversal of the A-scan peak at a frequency measured by the RF spectrum analyzer ~780 MHz, shown by the DSR vertical dashed line placed slightly above 11 mm. The M-method is functional well above this second limit, reaching over 14 mm in depth.

In conclusion, attenuation to half is obtained for the M-method and CMS methods at 8 mm, whilst for the D-method at 4.2 mm. Between the limits, C and DSR, conventional OCT technology can operate with software based resampling methods. However, the M-method extends the axial range to more than 40% than the limit set by the DSR. The better sensitivity achieved by the CMS in comparison to the D-method is due to using calculated masks of constant and top hat profile amplitude, i.e. of no spectral variation. The experimental mask provided by the D-method listens to the envelope of the tuned spectrum of the swept source.

The sensitivity for all three methods is measured at an OPD_M_ determining ~11 MHz pulsation, with a power of 1.6 mW on the sample and using the broadband mixer. Using a mirror as sample and applying the method explained in previous reports [1], CMS-methods sensitivity reached 85 dB. Comparatively, using the D-method and the M-method, sensitivity was 76 dB and 70 dB respectively. We expect better sensitivity to be achievable at larger OPD values, where higher cut-off high pass filters can be used to attenuate further the low frequency components due to the laser intensity modulation traversing the Down-converter.

When using the M-method with simple mixers, the signal at their output exhibits fluctuations due to the variation in the phase difference of the two signals, from the two interferometers, that can uncontrollably vary and also cross odd multiples of 90°. In the measurement of signal, we have used the fast, transversal scanner in the scanning head GXY, driven with a sinusoidal signal of 50 mV amplitude, at 1 kHz, just enough to create a little phase change. This was not necessary when using the I&Q demodulators. Also, this is not a problem when scanning, tiny lateral shifts of the beam moves it through height discontinuities and scattering features that create sufficient phase alterations. This was a specific behavior, used in the *en-face* time domain OCT, where no phase modulator was needed to create images [11,14].

### 3.5 Axial resolution

**Fig. 5:**
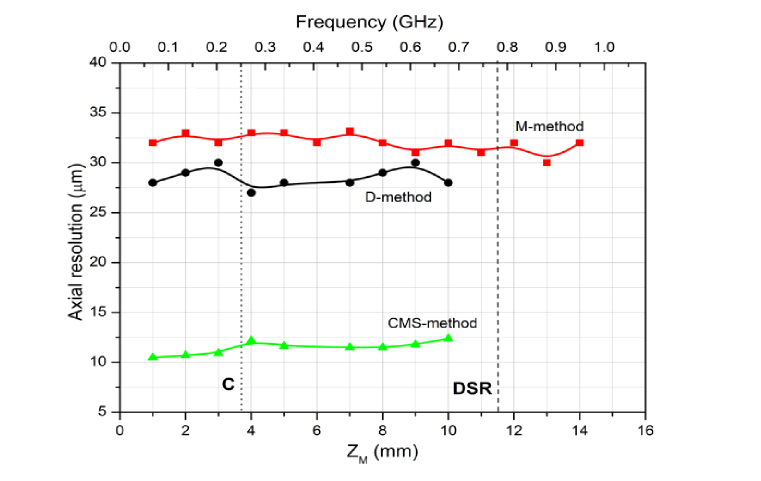
Axial resolutions produced by the M-method with the Broadband Mixer, D-method and the CMS-method using the 200 kHz SS

The evolution of the axial resolution with OPD is shown by the graphs in Fig. 5

. The axial resolution oscillates between 28 and 30 µm, with the D-method and 30-33 µm with the M-method using the broadband mixer. When using the M-method, LPF1 and LPF2 after the mixer were adjusted to 1 MHz Some reduction in intensity was noticed when the low pass filter cut-off was reduced below 300 kHz, as expected. However, the CMS procedure gives much better axial resolution, 10 µm (similar to what was also obtained using FFT applied to data resampled).

The axial resolution obtained is less than the expected value as determined by conventional FFT method and CMS using calculated masks. This is partly attributed to some unbalanced dispersion between the two interferometers. This can be seen as an oscillation of the photo-detected signal during sweeping for OPD = 0. In this configuration, the Master Interferometer must have similar dispersion parameters as the Slave one. In the first term in Eq. (5), if, Δ*h* ≠ 0 the temporal variation of the signal takes place even at Δ(OPD) = 0. The CMS method is superior to the D and M-methods in this respect, as the same interferometer is used at both Master and Slave stages, where the masks calculated incorporate the dispersion in the used interferometer. In this way, even if there is unbalanced dispersion in the interferometer, the CMS operation is not affected. However, it would be feasible in practice that the two interferometers are assembled with similar amount of unbalanced dispersion. This can be achieved by using similar components in both interferometers and does not represent an important disadvantage. Another reason for the lower axial resolution is the sampling duration. This was shortened below that of the laser sweeping duration to enhance the rejection of signals from outside the coherence gate represented by Δ(OPD) = 0.

The reason method D still gives worse axial resolution than the CMS using stored masks is due to two factors: (i) there is some dispersion left uncompensated in the Slave interferometer and (ii) the generated mask using the Master interferometer follows the envelope of the swept source spectrum, i.e. the final axial resolution is determined by two Gaussian profiles multiplied instead of being determined by a single Gaussian function as in CMS. This was also a disadvantage of first MS report [1], using experimentally acquired masks.

The effect of the narrower band of the I&Q demodulator, whose datasheet recommends 9-11 MHz was also considered. We evaluated how the axial resolution varies with the dominant frequency in the spectrum of the RF signal determined by the Master interferometer. The axial resolution was only a few microns worse with the I&Q demodulator than using the broadband mixer. This shows that the limited bandwidth of the I&Q demodulator is not the prime factor in determining the axial resolution, at least for the shallow depth position that determines 10 MHz modulation, where according to Fig. 2(a), the spread of frequencies in the RF spectrum is limited. The lower sensitivity of the M-method than in the state-of the-art OCT is due to noise in the signal spectrum outside the coherence gate.

We have also considered the effect of lower sampling rates in the M-mode than the 1.8 GS/s required by the D- and CMS-methods. The digitizer used for this study requires a minimum of 256 points to sample each spectrum, hence for a 3 μs interval, minimum sampling rate is 85 MS/s. Similar axial resolution values and decay of sensitivity with OPD curves were obtained for 100 MS/s, 250 MS/s and 1.8 GS/s. Each time the sampling rate was changed, the number of sample points was also altered as well as the delay provided by the Variable Delay generator.

### 3.6 Tolerance in the variation of the tuning curve

**Fig. 6:**
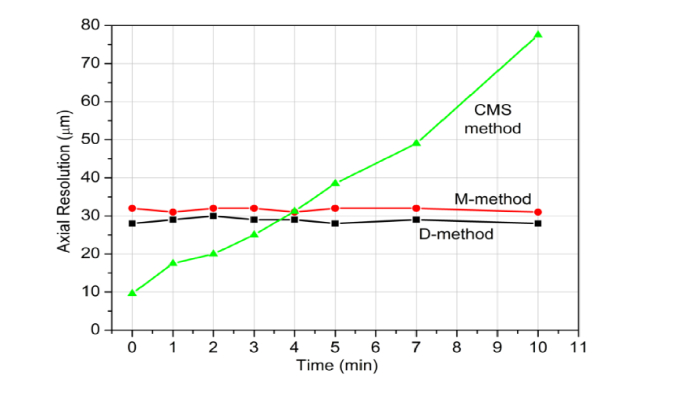
Temporal evolution of the axial resolution for the CMS, D and M-method using the broadband mixer over 10 minutes after the swept source was switched on. The data was evaluated for Z_M_ = 3 mm.

Using the 200 kHz swept source without its k-clock, it was noticed that the phase variation over its tuned spectrum varied in time in terms of minutes. Therefore, any software method using data resampling to produce A-scans fails in long run and recalibration is required. This study is not about a rigorous evaluation of this instability, but in demonstrating that the configuration in Fig. 2 can tolerate such variations. First, we investigated the instability and noticed that the axial resolution interval can double in matter of tens of minutes, on some occasions even in terms of several minutes. Figure 6

shows one of the variation curves of the axial resolution using the CMS, D and M-methods for a given OPD value. The CMS requires calculation of masks that depends on the stability of the phase variation. In this respect, the CMS method suffers exactly as the conventional software method based on FFT and the achievable axial resolution varies wildly in 10 minutes after switching on the swept source. On the contrary, the D and M-methods exhibited constant values for the axial resolution, i.e. the mixing method with masks created in real time by a Master interferometer tolerates instabilities in the tuning curve of the swept source. Left for longer, the CMS axial resolution recovers and then deteriorates again (not shown).

### 3.7 Images

To explore the M-method capabilities, *en-face* OCT images were produced. To simplify the comparison between the different modes of operation, we maintained the same scanning settings in all modes, as required to provide real time *en-face* display using the CMS method. To enable sufficiently fast display, the horizontal size of the image is set to N_x_ = 200 lateral points (along horizontal X-axis) scanned by the fast lateral galvo-scanner in the XY scanning head. Considering the sweeping rate of 200 kHz of the swept source, this gives 1 ms for each ramp of the triangular wave-form used to drive the fast galvo-scanner. CMS and D calculations are performed line by line in the 1 ms interval of the ramp not being used. Using 200 lines in the *en-face* image, a frame is acquired at 2.5 Hz by driving the vertical scanner with a saw-tooth ramp. In all modes, the computation time to produce a line in the *en-face* view is short. A benchmarking on a computer equipped with a processor Intel I5-4590 (4 cores) showed that when the spectrum is sampled into 1024 sampling points, using the CMS method, one line in the image can be produced in 65 µs while with the M-method in 50 µs. If only 256 sampling points are used, CMS can generate a line in the image in 20 µs while the M-method in 15 µs. The fact that the computation times of the two methods are similar is expected as they involve comparable number of mathematical operations. When a long axial imaging range is needed, for the D and CMS methods, the digitizer must sample data at a sampling rate as high as possible, in our case 1.8 GS/s. To sample 3 μs, 5376 points must be used to digitize the spectrum. The CMS procedure of producing a line in the *en-face* view takes 325 µs in this case. However, for a channeled spectrum corresponding to a large OPD value, the number of points needed scale with the down-conversion factor introduced above. For the swept source used here, DCo ~2x10^3^ but for longer coherence length swept sources DCo can be much larger, in which case the time required for CMS and D calculations become significant.

**Fig. 7:**
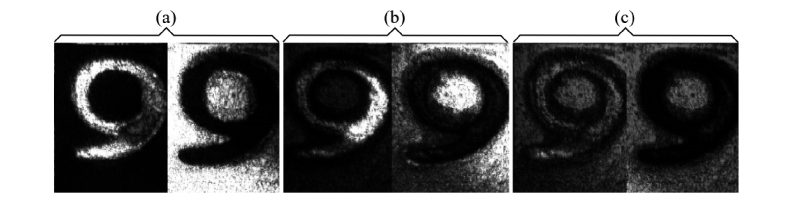
*En-face* OCT images obtained from a tilted 5-pence coin using the CMS-Method, distance between images 50 µm. (a) Z_M_ = 0.5 mm, (b) Z_M_ = 5 mm, (c) Z_M_ = 9.5 mm. Size: 2 x 2 mm^2^

For comparison, CMS method is used to display images from a 5-pence coin, as shown in Fig. 7

. The coin is slightly tilted and therefore the coherence gate intercepts fragments of the coin surface. Two images are shown, with a distance between axial positions of 50 µm, measured at: (a) z_M_ = 0.5 mm, (b) z_M_ = 5 mm, (c) z_M_ = 9.5 mm. The image in Fig. 7(a) corresponds to a depth within the normal axial range of the conventional SS-OCT using the clock in the swept source. The pair of images in Fig. 7(b) corresponds to an image obtained by the conventional technique using resampling, as the frequency generated in the photo-detected signal is outside the range covered by using the swept source clock. Its contrast is lower and even lower is the contrast for the pair of images in Fig. 7(c), close to the DSR limit. The decay of image contrast follows the decaying curve of sensitivity with OPD in Fig. 3.

**Fig. 8:**
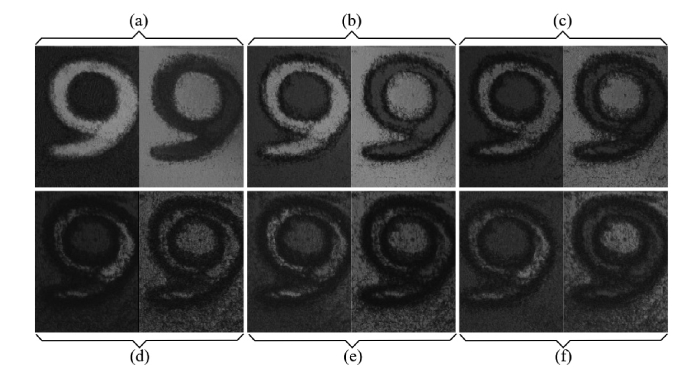
*En-face* OCT images obtained from the same tilted 5-pence coin using the M-Method, distance between images 80 µm, for Z_M_ = (a) 0.5 mm, (b) 5 mm, (c) 9.5 mm, (d) 12.5 mm, (e) 14 mm, (f) 16.5 mm. Image size 2 x 2 mm^2^.

The pair of *en-face* images of a 5 pence coin (Fig. 8

) are obtained using the M-method employing the broadband mixer. For better distinct between the images in each pair, the differential distance was increased to 80 µm. For the same Z_M_ values, the contrast reduces less, in accordance to the M graph in Fig. 4. The images in Fig. 8(b) correspond to the M-method applied to an OPD value exceeding the value limited by the swept source clock. The images in Fig. 8(c)-8(f) correspond to an OPD above the DSR. Here the D and CMS methods cannot be used, due to the limited digital sampling rate. Images are still visible from Z_M_ = 14 mm.

**Fig. 9:**
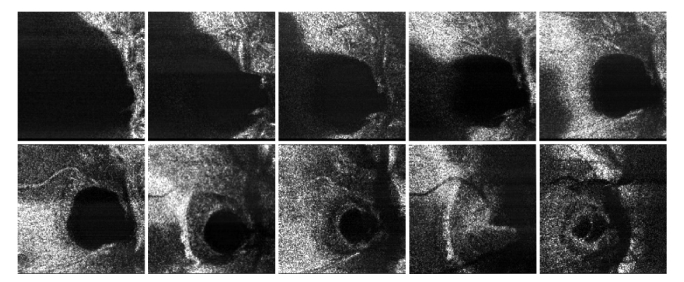
*En-face* OCT images of human optic nerve, collected from different axial positions. Lateral scan size ~4 mm x 4 mm.

A too low LPF frequency cut-off smears the *en-face* images, affecting their transversal resolution. By blocking any of the arms of the master interferometer, the OCT image displayed becomes dark. The configuration was then used to produce *en-face* OCT images from the eye and skin of a volunteer and the M-method and the LabV/IQ program. The power to the eye’s cornea and skin was 1.6 mW. An RF Mini-Circuits amplifier ZHL-32A + inserted between the balanced photodetector in the slave interferometer SBPD and the Down-converter improved the brightness in the images. Several images are shown in Fig. 9

from the optic nerve. As no fixation procedures were used, the images show different slices along the retinal tissue depth. No averaging or other techniques to improve the signal-to-noise ratio were applied.

Then, in Fig. 10

**Fig. 10:**
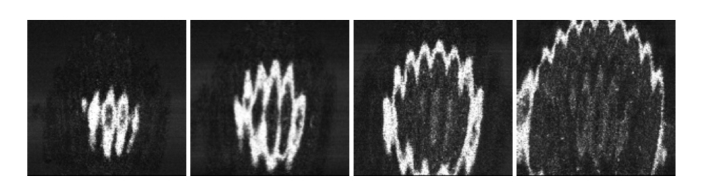
*En-face* OCT images obtained from a thumb, lateral scan size ~3 mm x 3 mm.

, images from skin finger are shown. Stratum corneum and epidermis are visible. If tissue is tilted, then the information acquired in the *en-face* view acquires aspects of cross section slices. This can be used to produce a look-alike cross section having an ultra-fast system delivering a single *en-face* OCT image based on the M-method.

## 4. Conclusions

A novel practical implementation of Master Slave interferometry is presented where Mask signals are generated in real time while sweeping the frequency of the swept source. A separate interferometer is employed, operating as a Master Interferometer, that generates the mask signal. This imposes a coherence gate while laterally scanning the beam over the sample in the Slave interferometer. The coherence gate so created leads to an operation similar to that reported in *en-face* time domain OCT, with the difference that here spectral domain principle is employed: (i) spectra are measured and mixed and (ii) signal is acquired by working away from OPD = 0 in each interferometer. In opposition to time domain OCT, where the coherence gate is established by OPD = 0, here the coherence gate condition is |OPD_M_ - OPD_S_| = 0.

The configuration based on two interferometers allows down-conversion of multi MHz - GHz radio frequency signal delivered by the photodetector unit. Down-conversion is demonstrated using two different types of down-converters. Using a broadband mixer, exploration of the depths in the sample is performed by changing the OPD in the Master interferometer. In this case, the expected decay of sensitivity with OPD is obtained, as with any SS-OCT configuration, determined by the overlap of the lengths of the two-interfering wave-trains (determined by the inverse of the dynamic coherence length of the swept source). When a narrowband I&Q demodulator is used, the frequency content of photo-detected signals needs to be kept constant. In this case, the reference arm length of the Slave interferometer is varied and by doing so, the sensitivity is maintained constant over the depth (similar to time domain OCT), apart from the attenuation due to the sample material. The level of sensitivity is however that as determined by the same dynamic coherence length of the source for the value of the OPD that determines the central frequency of the I&Q demodulator. Such a method should achieve better SNR, as the noise bandwidth is smaller. I&Q demodulators require applying a 90° phase to one of the signals, that limits their working bandwidth. Their limited bandwidth may reduce the optical band processed, due to the associated chirp. For comparison with the CMS method using generated masks, results are obtained using digitization of the photo-detected signals at the two outputs of the two interferometers, D-method. The M-method exhibits a lower decay with OPD than both the D-method and CMS- method when varying the OPD in the Master interferometer.

We envisage that the main advantage of the M-method is that it can preserve real-time operation irrespective of the sweeping rate. Conventional A-scan production can only be performed in real-time up to a few MHz, with some variation determined by the number of sampled points and dynamic range. For ultra-high tuning rates, fast digital oscilloscopes are used with memory. However, producing a single or a limited number of *en-face* OCT images in real-time, irrespective of the sweeping rate, whatever fast, becomes possible by using the MS method presented here employing two physical interferometers. The down-conversion method may become useful in the context of progress in the tuning speed of swept sources [19]. By applying time stretch technology, tens of MHz sweeping rates became available [20]. For such high rates, the MS configuration with two physical interferometers would prove useful. Even if a single measurement of distance is performed or even if only a single *en-face* OCT image is delivered, this becomes extremely useful in guiding the acquisition when using ultra-fast digital technology, with ultra-fast oscilloscopes and data stored. A single *en-face* OCT image displayed in real-time may deliver that necessary guidance to position the sample quick in the right place in terms of its lateral position, tilt and axial distance from the instrument. Also, assemblies of 2 or 4 Master interferometers may not look prohibitive, to deliver 2 or 4 *en-face* images from 2 or respective 4 depths simultaneously. It should be noticed that increase in the number of depths investigated does not require division of optical signal from the sample, only division of optical source power towards more interrogating (Master) interferometers. For example, the commercial Axsun swept source used here of only 15 mW could cope with more than two Master interferometers, considering that the reference power in a single interferometer is always attenuated by a factor larger than 2 to avoid saturating the photo-detector.

The main penalty in terms of cost in multiplying the number of Master interferometers is the photodetectors. Considering that tens of MHz sweeping rates with N > 1000 lead into tens of GHz bandwidth, such fast photodetectors may reach tens of thousands of US dollars. However, this may be worth bearing for the provision of real time imaging, with larger powers to the sample, while still observing the limits set by safety. The configuration presented down-converts the bandwidth of the signal to be processed to the level of the sweeping rate, allowing real time display of an *en-face* image or measurement of a distance. In addition, the cost of the optoelectronic processor (interferometer and Down-converter) is much less than that of a fast digitizer or fast oscilloscope. Even if a fast-digital oscilloscope can sample many tens of GS/s, the data acquired is too large to be transferred for calculation and usually data is stored, preventing real time display of the images. In comparison with 3D detailed collection of data using either FT or CMS based OCT, where the 3D data allows subsequent flattening of tissue and selection of *en-face* layers belonging to a certain structural tissue layer, this is not possible using the M-method shown here. The *en-face* OCT image is that collected at the OPD dictated by the Master interferometer and therefore if the tissue is curved or the surface presents curvature, then the displayed image contains structure from several neighboring layers in depth.
